# Foreign bodies in children's lower urinary tract: A case series and literature review

**DOI:** 10.3389/fped.2022.1095993

**Published:** 2023-01-10

**Authors:** Tongshuai Kuang, Wei Cai, Weite Qian, Xiaokun Lin

**Affiliations:** ^1^ Department of Pediatric Surgery, The Second Affiliated Hospital and Yuying Children's Hospital of Wenzhou Medical University, Wenzhou, China; ^2^ Wenzhou Key Laboratory of Children Genitourinary Diseases, The Second Affiliated Hospital and Yuying Children's Hospital of Wenzhou Medical University, Wenzhou, China

**Keywords:** lower urinary tract, foreign bodies, children, surgery, cystoscopy

## Abstract

**Background:**

Children with foreign bodies (FBs) in the lower urinary tract have rarely been reported, and their management remains challenging. This study aimed to describe the characteristics and treatment of FBs in children's lower urinary tract.

**Methods:**

We retrospectively analyzed the clinical data on lower urinary tract FBs that were removed in our hospital from August 2017 to August 2022, including demographics, location, symptoms, imaging examinations, and treatment.

**Results:**

Four male patients were enrolled, whose ages ranged from 9 to 13 years, with a mean age of 11 years. The course of the disease ranged from 3 h to 2 weeks. Their imaging characteristics were reviewed and analyzed, and two FBs were located in the bladder and two in the urethra. Mosquito forceps were used to remove an acne needle through the urethra in one case. Cystoscopy was first attempted in three cases, in only one of which was the FB removed successfully under endoscopic minimally invasive surgery. In the remaining two cases, removal *via* transurethral cystoscopy failed, whereby leading to cystotomy being performed. The FBs comprise a skipping rope, hairpin, magnetic bead, and acne needle. The postoperative recovery was uneventful, and no complications occurred during the follow-up period of 3 to 6 months.

**Conclusion:**

It is rare for children to have FBs in the lower urinary tract. An early diagnosis, as well as appropriate management of lower urinary tract FBs, can significantly reduce complications. Surgical removal of lower urinary tract FBs can be safe and effective, and relatively better outcomes can be achieved.

## Introduction

Foreign bodies (FBs) are an uncommon condition in the lower urinary tract of children, especially in the urethra and bladder. FBs in the lower urinary tract are most commonly found in adolescents and are usually inserted by the persons themselves to satisfy sexual desires or curiosity (1). The presence of FBs in the lower urinary tract can result in symptoms such as dysuria, hematuria, increased frequency and urgency of urination, and pain in the lower abdomen and pelvis. Moreover, these symptoms depend on the nature, shape, size, mobility, location, and residence time of the FBs. Pediatric patients have reported FBs in the form of wooden sticks, plastic pens, screws, erasers, pins, electrical wires, jewelry, pencils, and household items (2–7). Furthermore, FBs in adults can be in the form of cotton swabs, tampons, clips, pen casings, straws, batteries, earphone wires, and nail scissors (8–11).

An early diagnosis and prompt removal of FBs constitute effective treatment with which to avoid potential complications. However, it is always difficult to obtain accurate medical histories from pediatric patients with this condition, especially those who insert objects for sexual pleasure (9). So far, few studies have investigated FBs located in the lower urinary tract, which is rare in children. To improve clinical management of children with FBs in the lower urinary tract and reduce the risk of complications, we retrospectively analyzed a series of four pediatric patients with FBs in the lower urinary tract who were treated surgically at our institution. It is helpful for pediatric surgeons to summarize the clinical characteristics and treatment experiences to raise awareness of the disease for an early diagnosis and intervention.

## Materials and methods

Between August 2017 and August 2022, 4 patients underwent treatment for FBs in the lower urinary tract at our center. We retrospectively reviewed and analyzed the records of these patients to characterize the nature of the FBs, each patient's clinical presentation, and the management of the FBs (Table 1).

**Table 1:** Characteristics of the lower urinary tract foreign bodies and their management.

Case	Age (Years)	Clinical presentation	Duration	Location	X-Ray	US	CT	Retrieval method	Characteristics
1	11	Recurrent dysuria and hematuria	2 weeks	Bladder	Not found	Enhanced echoes	Hyperdense lesion	Cystoscopy conversion to open cystotomy	Skipping rope
2	9	Urethral pain	10 h	Urethra	High density shadow	Not done	Strip density shadow	Mosquito forceps	Acne needle
3	11	Lower abdominal pain	1 day	Bladder	High density shadow	Not done	Irregular hyperdense shadow	Cystoscopy conversion to open cystotomy	Multiple magnetic beads
4	13	No symptoms	3 h	Urethra	High density shadow	Not done	Not done	Cystoscopy	Hairpin

## Case presentation and results

**Figure 1:**
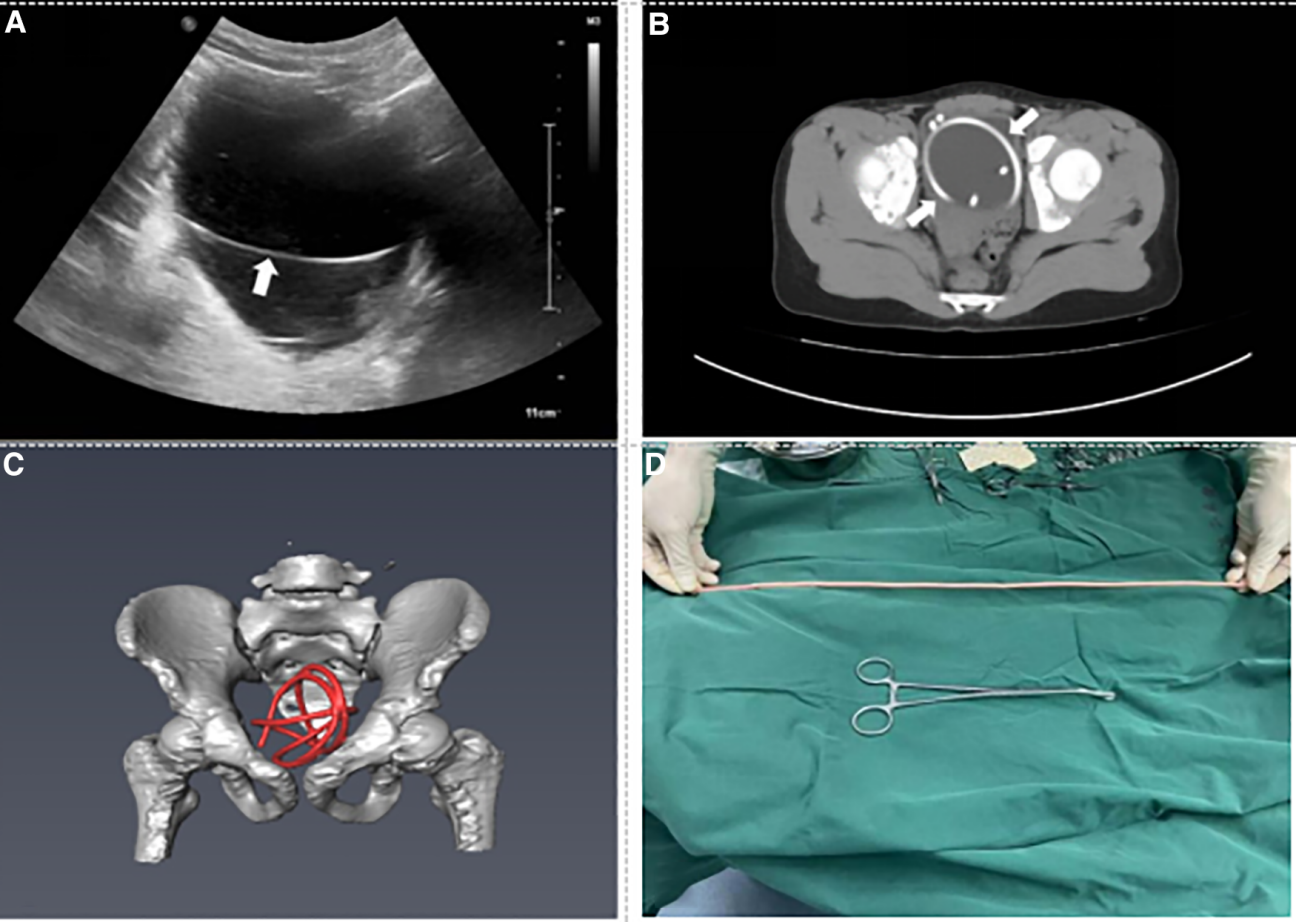
Preoperative imaging data and foreign body. (**A**) US shows the bladder foreign body (see arrow). (**B**) CT of the pelvis shows the crooked foreign body in the bladder (see arrow). (**C**) Three-dimensional reconstruction CT shows the lumpy foreign body in the bladder (red mark). (**D**) Foreign body is a skipping rope about 50 cm long.

Case 1: An 11-year-old boy presented to the emergency department with repeated dysuria with hematuria, which had begun two weeks prior. He had been previously brought to a local hospital by his family and had been diagnosed with a urinary tract infection. However, he had not improved significantly after oral antibiotic treatment. Physical examination was unremarkable, with the exception of mild suprapubic tenderness. Routine urine examination revealed that white and red blood cells were elevated, 1333.00/μl and 390.00/μl, respectively. Ultrasound (US) examination identified enhanced echoes in the bladder (Figure 1A). A pelvic computed tomography (CT) scan displayed an extremely hyperdense lesion in the bladder (Figure 1B). Three-dimensional reconstructed images using Amira software emphasized that an intravesical FB could be considered (Figure 1C). An attempt was made to insert a cystoscope to remove the rope under general anesthesia, but it failed due to the smooth surface of the FB. Afterward, cystotomy was performed, and the FB was removed (Figure 1D). The operation time was about 1 h. Intraoperative findings depicted that the FB was a bent and winding rope, about 50 cm in length. The patient received anti-infective treatment after surgery and was followed up for three months after being discharged, and no complications occurred.

**Figure 2:**
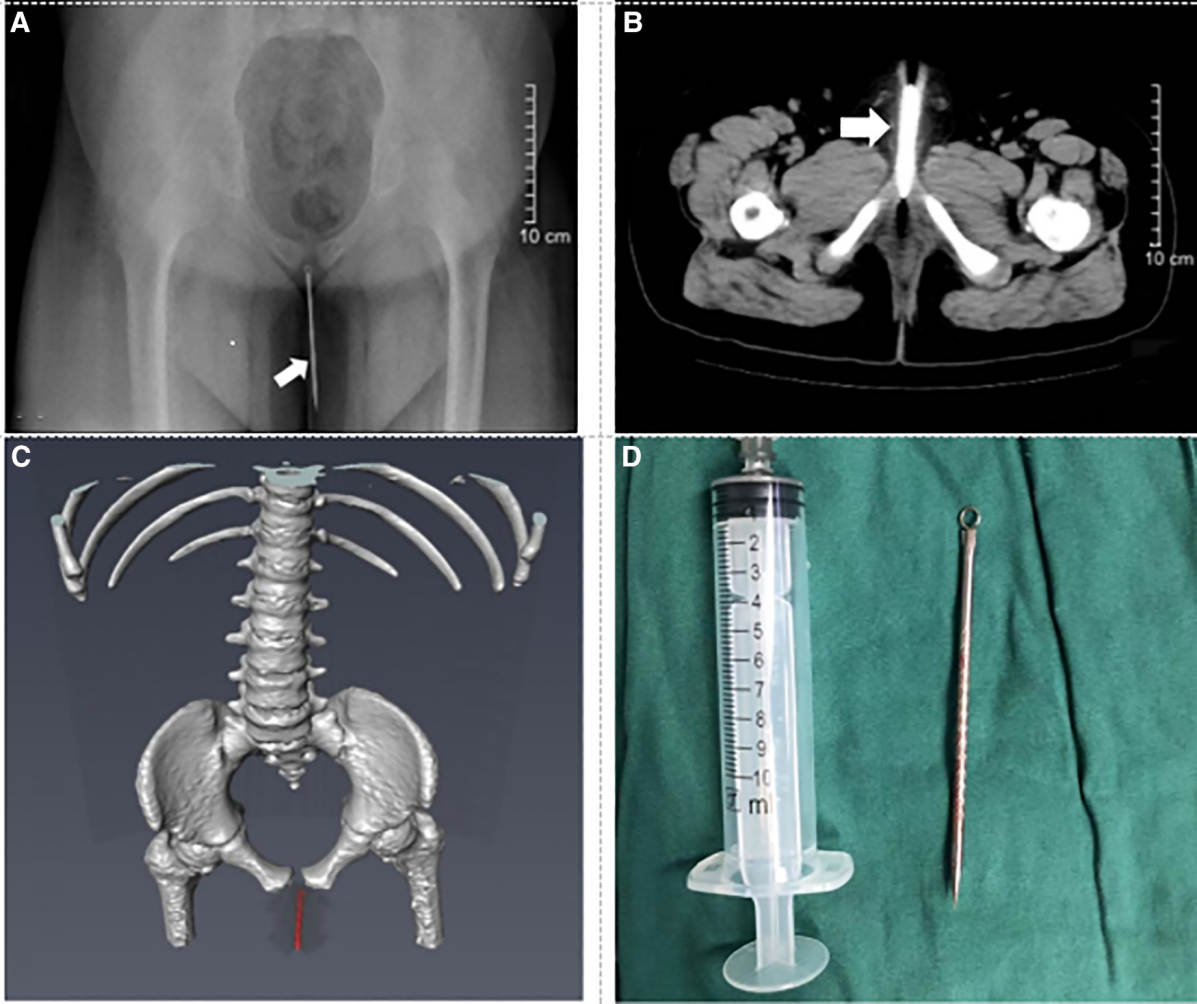
Preoperative imaging data and foreign body. (**A**) Plain film x-ray shows the urethral foreign body (see arrow). (**B**) CT of the pelvis shows the straight foreign body in the urethra (see arrow). (**C**) Three-dimensional reconstruction CT shows the straight foreign body in the urethra (red mark). (**D**) Foreign body is an acne needle about 10 cm long.

Case 2: A 9-year-old boy presented to the emergency department complaining about urethral pain 10 h prior. A vertical x-ray film of the abdomen demonstrated a needle-like FB (Figure 2A). CT of the lower abdomen demonstrated a strip and dense shadow in the cavernous region of the urethra (Figure 2B). Three-dimensional reconstructed images *via* Amira software emphasized that a urethral FB could be considered (Figure 2C). The patient underwent surgical treatment under general anesthesia. Mosquito forceps were inserted into the urethra to clamp the proximal end of the FB, and a metal needle (about 10 cm in length) was successfully removed from the urethra (Figure 2D). The operation time was about 5 min. The patient was discharged on the following day. There were no complications in the late follow-up.

**Figure 3:**
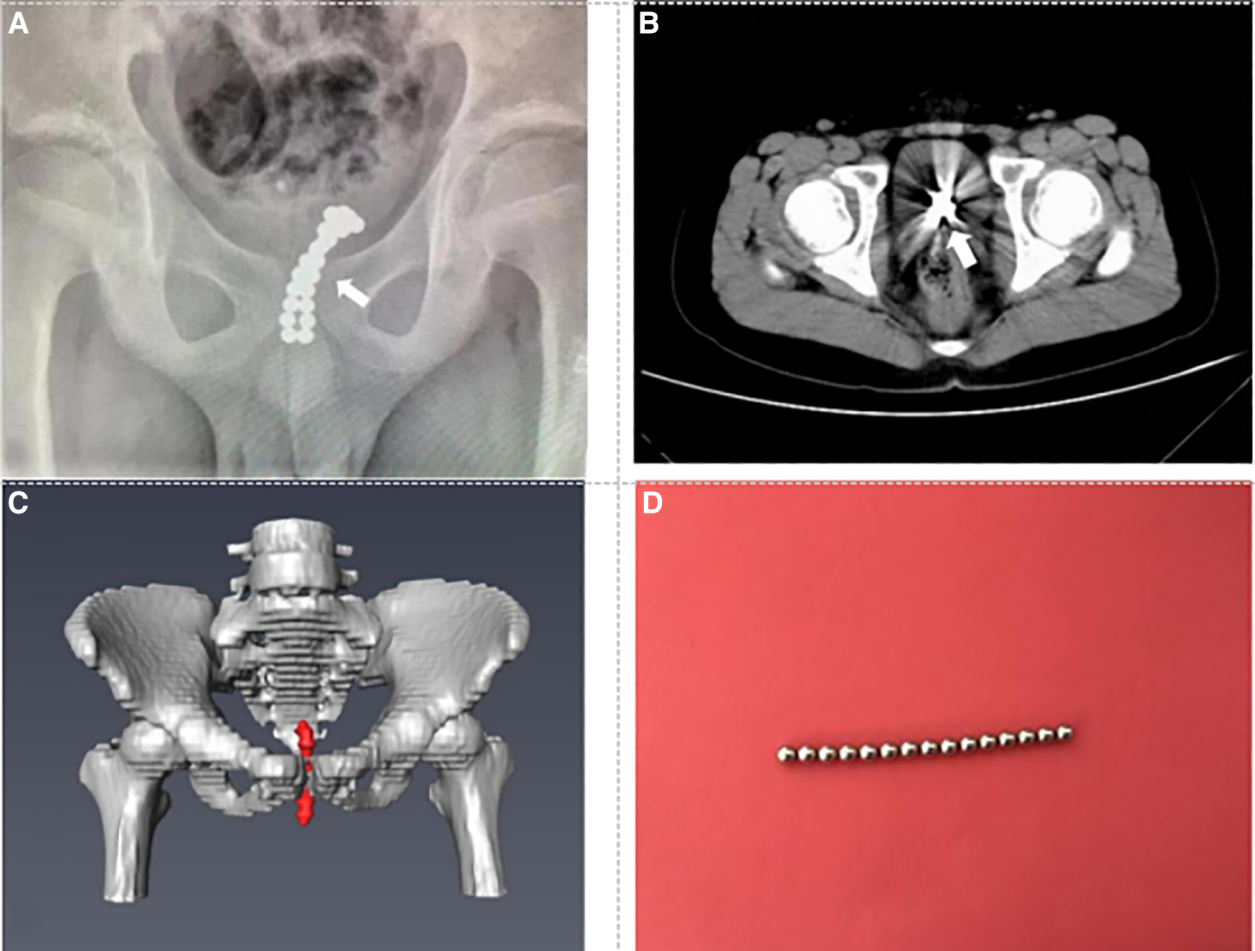
Preoperative imaging data and foreign body. (**A**) Plain film x-ray shows multiple small spherical foreign bodies in the bladder (see arrow). (**B**) CT of the pelvis shows the massive foreign body in the bladder (see arrow). (**C**) Three-dimensional reconstruction CT shows the stripe foreign body in the bladder (red mark). (**D**) Foreign bodies are multiple magnetic beads (partly).

Case 3: A 11-year-old boy presented to the emergency department complaining of lower abdominal pain 1 day ago. A vertical x-ray film of the abdomen showed a strip-shaped image of FBs (Figure 3A). CT of the lower abdomen showed irregular hyperdense shadow in the bladder (Figure 3B). Three-dimensional reconstructed images *via* Amira software emphasized that intravesical FBs could be considered (Figure 3C). An attempt was made to insert a cystoscope to remove the FBs under general anesthesia but failed because the grasp forceps were attracted by the magnetic FBs. Then, a cystotomy was performed, and the FBs were removed (Figure 3D). The operation time was about 50 min. The patient received anti-infective treatment after surgery and was followed up for six months after being discharged, and no complications occurred.

**Figure 4:**
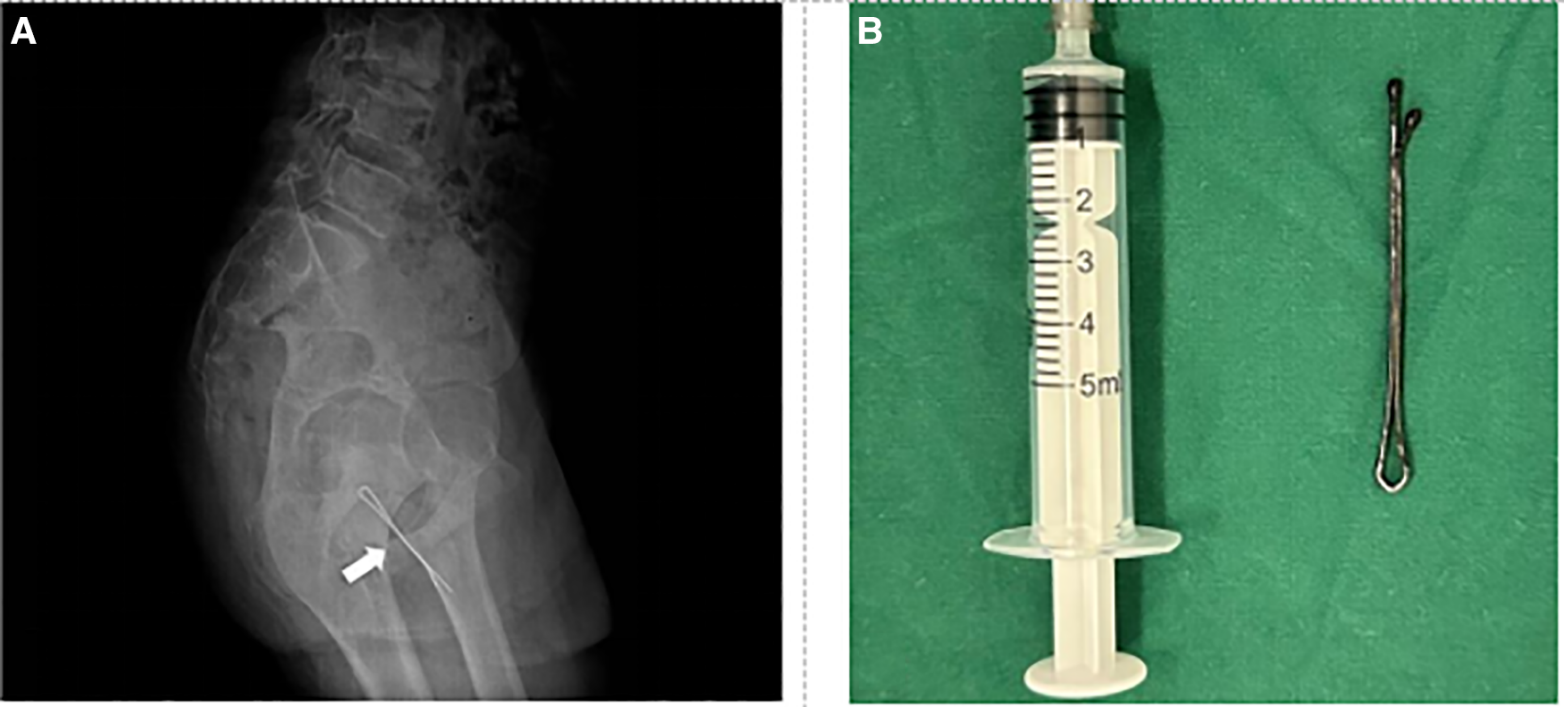
Preoperative imaging data and foreign body. (**A**) Plain film x-ray shows the urethral foreign body (see arrow). (**B**) Foreign body is a hairpin about 6 cm long.

Case 4: A 13-year-old boy presented to the emergency department complaining of inserting the hairpin into the urethra 3 h ago without any symptoms.A vertical x-ray film of the abdomen showed a hairpin-shaped image of FB (Figure 4A). Physical examination revealed a FB in the posterior urethra. The patient underwent transurethral cystoscopy to extract the FB by using forceps. The operation time was about 10 min. The FB was a 6 cm hair clip (Figure 4B). The patient was discharged the next day. There were no complications in the late follow-up.

## Discussion

The presence of an FB in the lower urinary tract of children has been an interesting topic in representing a management challenge. Although numerous cases of self-inflicted FBs in the lower urinary tract have been reported in boys, they have also been reported in girls (2). In our series, all four cases were of male children. Among children, the most common motive behind inserting an FB into the lower urinary tract is simple curiosity. During childhood and inherent to their development, children explore their bodies and orifices (12). Other causes include psychiatric disorders and sexual stimulation (13).

A diagnosis is made using history-taking and clinical examination. However, children who themselves insert FBs into the lower urinary tract may be hesitant to provide this information for fear of embarrassment or scolding. They will not tell their parents or doctors until they have clinical symptoms. Clinical presentations of FBs in the lower urinary tract may vary from being initially asymptomatic to complaints of dysuria, hematuria, frequency, poor stream, and urinary retention. Some children will initially try to remove these FBs themselves. However, this often results in FBs being inserted deeper as well as aggravating damage (14, 15). Once FBs are retained in the lower urinary tract for long periods, some patients develop serial complications such as recurrent urinary tract infections, stone formation, urethral fistula, urethral stenosis, or even urosepsis (16). In our series, case 1 did not provide an accurate medical history in the local hospital, which led to a delay in the diagnosis. The condition was not diagnosed in our hospital until there were repeated urinary tract infections.

It is essential to perform a preoperative imaging examination of FBs to plan for surgery. Due to its innocuous (non-ionizing) and noninvasive properties, US is considered the best imaging method for non-radiopaque FBs. Plain abdominal films are usually sufficient to locate and identify metallic and radiopaque FBs. However, in cases where the provider does not have access to US or is unable to visualize the FB, or wherein there is a concern of perforation, a CT scan may be obtained. For FBs in the lower urinary tract, CT examination and three-dimensional reconstruction are more precise and clearer in determining the specific location of the FBs. In our series, we have three patients who have undergone three-dimensional reconstruction and can see the location of the FBs.

The treatment of FBs in the lower urinary tract should be aimed at completely removing the FBs with minimal complications. Various treatment methods of removal have been described, including direct extraction, endoscopic treatment, open surgery, and laparoscopic treatment. For anterior urethral FBs, we can first try to directly use vascular forceps to remove the FBs, failing which, the endoscopic approach should be a choice for removing the FBs. Pediatric surgeons usually remove FBs through the use of grasping forceps guided by cystoscopy or ureteroscopy (17). For posterior urethra and bladder FBs, cystoscopy is the preferred method with which to remove the FBs, albeit depending on the shape, location, and severity of the injury. In children, however, the removal of FBs represents a great challenge, as the size of the pediatric urethra may hinder safe transurethral removal (18). Endoscopic treatment is effective in some cases; in other cases, meanwhile, endoscopic treatment fails because of the need to reduce urethral injury during transurethral removal of FBs or due to the difficulty of grasping FBs — open surgery is thus required (19). This includes suprapubic cystotomy for intravesical FBs and external urethrotomy for objects stuck in the penile urethra. In our series, there were also two patients whose FBs could not be removed under cystoscopy and who had to change to open surgery. Recently, with the development of minimally invasive technology, the laparoscopic approach for intravesical surgery using pneumovesicum has been widely used in the surgical treatment of urological diseases (20). Reddy reported a case of a child with Blu-Tack stuffed into the bladder (21).Utilizing a cystoscope as the optical device through the urethra, a 10 mm laparoscopic port was introduced under the vision for extraction of the complex FB while the bladder remained insufflated with carbon dioxide at a pressure of 12 mmHg. In this way, air cystoscopy can give surgeons a better view in cases in which vision is compromised under water-contrast cystoscopy.

**Figure 5:**
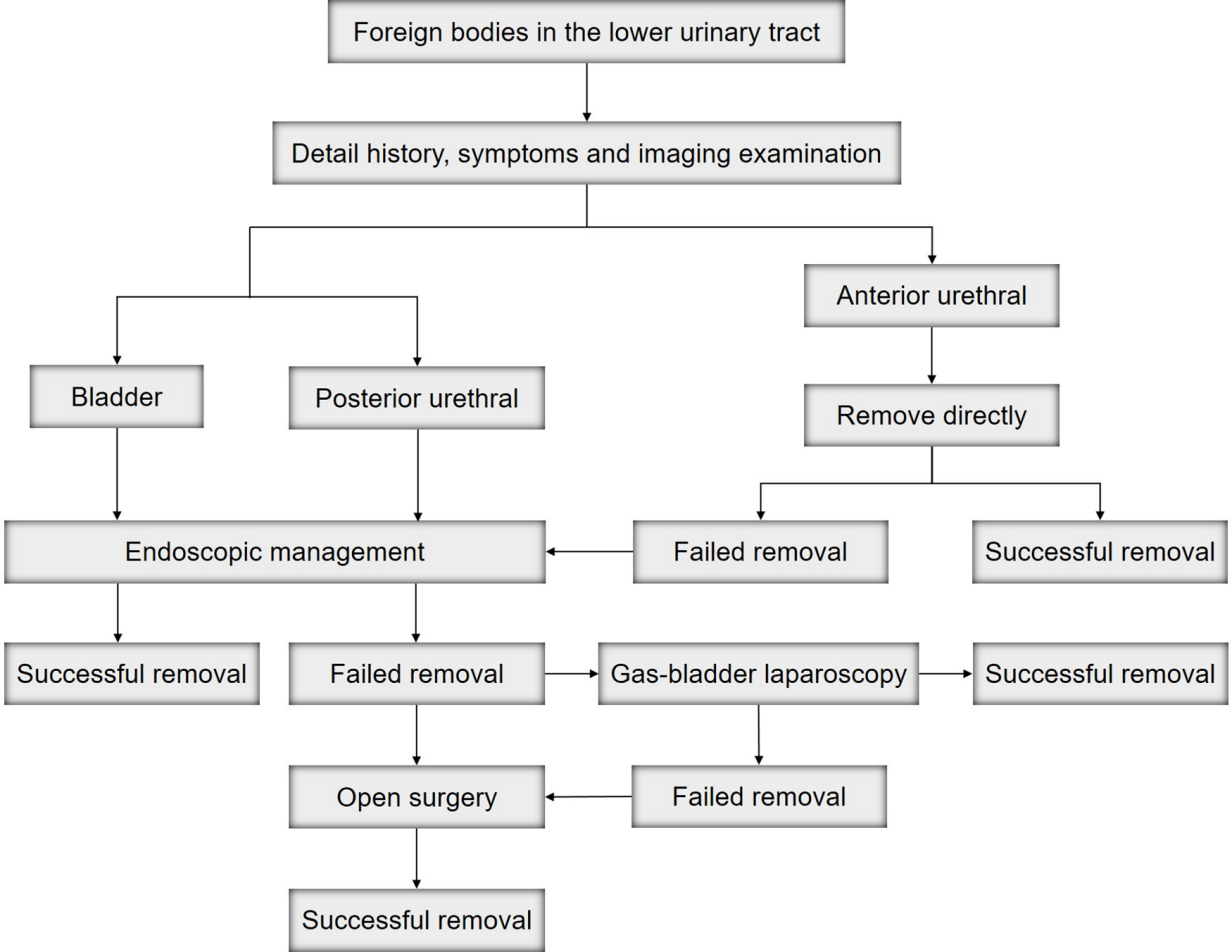
Algorithm for management of FBs in the lower urinary tract in pediatric patient.

Although the prognosis for these types of FBs is generally very good, early recognition of FBs in the lower urinary tract, as well as appropriate management are very important (Figure 5). Second, it is essential to develop primary preventative strategies for FB injuries. In our opinion, the education of parents and children might be an effective preventative method. Parents should not only guide children's behavior correctly but also pay attention to children's psychological conditions. The main limitation of this study was that it was a retrospective-descriptive review with few cases. Therefore, it is necessary to collect more cases or initiate a multicenter study in the future.

## Conclusions

Lower urinary tract FBs in children are clinically rare. Most of the time, they are difficult to diagnose because of an ambiguous medical history and can be easily overlooked. Therefore, a timely diagnosis and effective management of children's lower urinary tract FBs are of paramount importance in reducing further complications. Minimally invasive endoscopy remains the first-line approach to diagnostic removal of FBs in the lower urinary tract in pediatric patients. Open surgical removal may be performed in cases in which endoscopic techniques have failed. Surgical removal of lower urinary tract FBs can be safe and effective, and relatively better outcomes can be achieved. Education of parents and children might be an effective method of prevention.

## Data Availability

The raw data supporting the conclusions of this article will be made available by the authors, without undue reservation.
